# Decline in radioiodine use but not total thyroidectomy in thyroid cancer patients treated in the United Arab Emirates - A retrospective study

**DOI:** 10.1016/j.amsu.2021.102203

**Published:** 2021-03-04

**Authors:** Malik Azhar, Faisal Aziz, Salama Almuhairi, Mohammad Alfelasi, Ali Elhouni, Rizwan Syed, Humaid O. Al-Shamsi, Khaled M. Aldahmani

**Affiliations:** a Endocrine Division, Tawam Hospital, Al Ain, United Arab Emirates; b Departments of Medicine, College of Medicine and Health Science, UAE University, United Arab Emirates; c Division of Endocrinology and Diabetes, Medical University of Graz, Graz, Austria; d Center for Biomarker Research in Medicine - CBMed, Graz, Austria; e ENT Division, Tawam Hospital, Al Ain, United Arab Emirates; f Radiology Department, Tawam Hospital, Al Ain, United Arab Emirates; g Emirates Oncology Society, Dubai, United Arab Emirates; h College of Medicine, University of Sharjah, Sharjah, United Arab Emirates; i Burjeel Cancer Institute, Mohamed Bin Zayed City, Abu Dhabi, United Arab Emirates

**Keywords:** Thyroid cancer, Malignancy, Management, Radioactive iodine, Total thyroidectomy

## Abstract

**Objectives:**

To assess the trend of clinicopathological features and treatment modalities in patients with thyroid cancer (TC) in the largest oncology center in the United Arab Emirates (UAE).

**Methods:**

A retrospective analysis of patients with TC presenting to a tertiary care hospital in Al Ain, UAE between September 2008 and December 2018 identified using ICD 9 & 10 codes was performed. Data on demographics, histopathology, surgical extent, and use of Radioiodine (RAI) were extracted. Exact logistic and ordinal logistic regressions were performed to analyze the annual trend in features and management of TC, and logistic regression analysis was performed to identify predictors of total thyroidectomy and RAI use.

**Results:**

A total of 762 patients were included in the analysis (mean age: 39.6 ± 12.6 years, 45 (60%) women). The majority (92.2%) were diagnosed with papillary thyroid cancer (PTC) and 83.9% had tumor size of <4 cm. All patients underwent surgery (93.8% total thyroidectomy, 6.2% lobectomy) and 77.4% received RAI therapy overall with a significant (p < 0.001) decline from 100% in 2008 to 60% in 2018. In multivariate analysis, nationality, and lymph node (LN) involvement were significant predictors of total thyroidectomy, while nationality, LNs, year of diagnosis, and tumor size significantly predicted RAI use.

**Conclusion:**

Most patients in our cohort were diagnosed with localized PTC with no significant change in the extent of surgical approach but a substantial decline in RAI therapy administration over time. Nationality and LN involvement were significant predictors of surgical extent and RAI use.

## Highlights

• This is the largest study evaluating clinicopathological features and treatment modalities in TC patients in the UAE.
• Most patients were diagnosed with localized PTC.
• A substantial decline in RAI therapy administration but not the extent of thyroidectomy was observed during the study period.
• Nationality and LN involvement were significant predictors of surgical extent and RAI use.

## 1 Introduction

Management of thyroid cancer (TC) has significantly changed in the last decade with several guidelines advocating for a conservative approach in the majority of patients [1,2]. While these changes resulted in lower rates of total thyroidectomy and radioiodine administration in some parts of the world [3,4], data on TC from the Middle East and North Africa (MENA) region has largely focused on incidence, characteristics, and treatment outcomes with recent exploration of TC genetics [[5], [6], [7], [8], [9], [10], [11], [12]]. Little is known about the trend in TC management in this region.

It is difficult to predict if management of TC in the MENA region would follow similar trends compared to other parts of the world. For example, it is unknown if patients or even physicians are willing to observe micro papillary TC rather than offering surgery, at least lobectomy. Similarly, attitudes toward the extent of surgery and use of RAI in localized TC following the publication of the guidelines from the United Kingdom (UK) in 2014 and the 2015 American Thyroid Association (ATA) has not been well reported in the MENA region. Furthermore, ethnicity has been shown to influence the incidence and outcomes of TC in some studies [13,14]. Whether ethnicity impacts management of TC patients in our region remains unclear.

In the United Arab Emirates (UAE), TC represents the third most common malignancy in the population mostly occurring in the 3rd-4th decades of life and presenting as a localized disease [15]. We aimed to study trends of TC characteristics and treatment modalities in patients presenting to the largest oncology center in the UAE over the last decade. In addition, we evaluated factors associated with the extent of surgery and RAI therapy.

## 2 Materials and methods

### 2.1 Study setting

This study was conducted in Tawam hospital (TWM), which provides tertiary care services to patients in the UAE. It is the only center delivering radioactive iodine (RAI) therapy and radiotherapy in Al Ain city. Most TC patients are followed in designated TC clinics within the endocrinology division or less commonly through other clinics (surgery, radiation, or medical oncology). The hospital runs monthly multidisciplinary team-based discussions on patients with TC, wherein decisions regarding RAI treatment and dose selection are determined jointly by nuclear medicine physicians and endocrinologists.

### 2.2 Study design and participants

We retrospectively evaluated all patients with TC presenting to TWM during the period of September 2008 and December 2018. The beginning of data collection (September 2008) reflects the inception time of electronic medical records (EMRs) use in the hospital. Cases of TC were extracted from the EMRs using the International Classification of Disease 9 & 10 codes (193 & c73). Other variables were demographic information, year of diagnosis, type of TC, histopathological subtype, lymph node (LN) involvement, type of thyroid surgery, use of RAI treatment, and radiation therapy. The extent of surgery, the exact details of histopathology, and the RAI dose were retrieved from the respective specialty reports. Tumor staging was reported according to the 8th edition of the American joint committee on cancer (AJCC)/TNM staging system [16]. Those with incorrect pathological diagnosis, incomplete data, or noninvasive follicular type PTC were excluded.

This study was registered in the Chinese clinical trial registry (chiCTR2100043249) [17].

### 2.3 Ethical considerations

The study was approved by Al Ain Medical District Human Research Ethical Committee and consent was waved.

### 2.4 STROCSS compliance

This work has been reported in line with the STROCSS criteria [18].

### 2.5 Statistical analysis

The data were extracted using Microsoft Excel 2015 and imported into Stata 16.0 for statistical analysis. Continuous variables were described using means and standard deviations (±SD). Categorical variables were described as frequency distributions. Variables of clinical features and management were cross tabulated with the year of diagnosis and exact logistic and ordinal logistic regressions were performed to analyze the annual trend in features and management of TC as appropriate. The results of relative trend were reported as odds ratio (OR) with an overall p_trend_. The OR = 1 indicated no change over years, OR <1 indicated a decline, while OR >1 indicated an increase over years. Simple and multiple logistic regression analysis were performed to assess the unadjusted and adjusted association of management of TC (RAI, surgical management) with age, gender, nationality, year of diagnosis, tumor subtype, and other co-variates. The results of simple and multiple logistic regression were reported as odd ratios (OR) and adjusted odds ratios (AOR) respectively with their corresponding 95% confidence intervals (CI) and p-values.

## 3 Results

### 3.1 Overall patients’ demographics

A total of 762 patients were included in the analysis (Table 1). The mean age at the diagnosis was 39.7 (±12.6) years and 583 (76.5%) were women. The vast majority of patients (92.1%) were diagnosed with PTC. Of those, the histology subtype was available for 555 patients with the two most common being classical (73.5%) and invasive follicular variant (22.3%). About 83.8% of the patients (N = 555) had a tumor size of <4 cm at diagnosis. The stage of TC was documented in 590 patients. Of those, 523 (88.6%) patients were stage I while 23 (3.9%) were stage IV. All patients underwent surgery with the majority receiving (93.8%) total thyroidectomy and 566 (77.4%) RAI therapy.

**Table 1:** Characteristics of thyroid cancer patients (n = 762).

Demographics	N (%)/Mean (±SD)
Age – years (n = 762)	39.7 (±12.6)
Gender (n = 762)	
Female	583 (76.5)
Male	179 (23.5)
Nationality	
UAE	349 (45.8)
Others	413 (54.2)
Tumor size (n = 551)	
≤1 cm	147 (26.7)
1.1–1.9 cm	188 (34.1)
≥2–3.9 cm	127 (23.1)
≥4 cm	89 (16.1)
Tumor Type (n = 762)	
PTC	702 (92.1)
FTC	35 (4.6)
MTC	19 (2.5)
Anaplastic	6 (0.8)
PTC Subtype (n = 555)	
Classical	408 (73.5)
Follicular	124 (22.3)
Others	23 (4.1)
Stage (n = 590)	
I	523 (88.6)
II	35 (5.9)
III	9 (1.5)
IV	23 (3.9)
Type of surgery (n = 754)	
Hemithyroidectomy	47 (6.2)
Total thyroidectomy	707 (93.8)
RAI ablation (n = 731)	
Yes	566 (77.4)
No	165 (22.6)
Radiotherapy use (n = 762)	10 (1.3)

MTC: Medullary Thyroid Cancer, FTC: Follicular Thyroid Cancer, PTC: Papillary Thyroid Cancer, RAI: Radioactive Iodine, UAE: United Arab Emirates.

N does not always make up to 762 due to missing data for some variables.

### 3.2 Thyroid cancer characteristics over 10 years

**Fig. 1:**
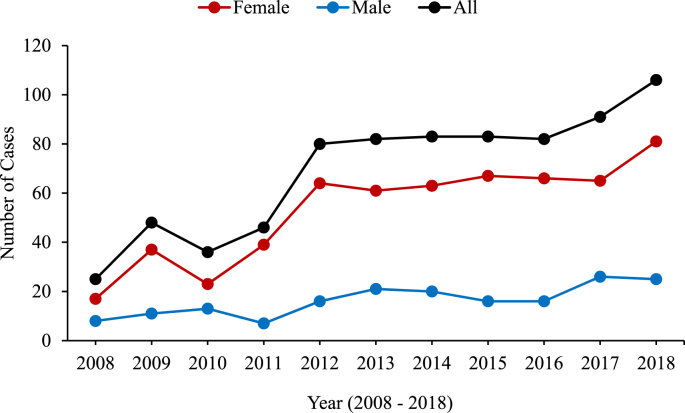
Incident cases of thyroid cancer between 2008 and 2018, overall and by gender.

The annual number of TC patients substantially increased during the study period from 25 cases in 2008 to 106 cases in 2018. Compared to men, the increment was significantly higher in women (Fig. 1). PTC was the most common type with a gradual increase (p = 0.674) from 88% in 2008 to 95% in 2018 (Table 2). Stage 1 Tumors represented the majority of TC patients in 2008 and also showed an insignificant (p = 0.267) increase till 2018. During the study period, compared to non-PTC types, the proportion of patients with PTC increased by 11% on average (p = 0.021). In contrast, patients presenting with advanced **TC thyroid cancer** relatively decreased by 10% (p = 0.022) compared to stage 1 cancer.

**Table 2:** TC cases over years, overall and by characteristics.

	2008	2009	2010	2011	2012	2013	2014	2015	2016	2017	2018	OR (P_trend_)
**Overall**	25 (3.3)	48 (6.3)	36 (4.7)	46 (6.0)	80 (10.5)	82 (10.8)	83 (10.9)	83 (10.9)	82 (10.8)	91 (11.9)	106 (13.9)	–
**Gender**												
Female	17 (68.0)	37 (77.1)	23 (63.9)	39 (84.8)	64 (80.0)	61 (74.4)	63 (75.9)	67 (80.7)	66 (80.5)	65 (71.4)	81 (76.4)	0.99 (0.777)
Male	8 (32.0)	11 (22.9)	13 (36.1)	7 (15.2)	16 (20.0)	21 (25.6)	20 (24.1)	16 (19.3)	16 (19.5)	26 (28.6)	25 (23.6)	
**Nationality**												
UAE	10 (40.0)	16 (33.3)	11 (30.6)	23 (50.0)	36 (45.0)	43 (52.4)	40 (48.2)	44 (53.0)	33 (40.2)	43 (47.3)	50 (47.2)	0.96 (0.155)
Others	15 (60.0)	32 (66.7)	25 (69.4)	23 (50.0)	44 (55.0)	39 (47.6)	43 (51.8)	39 (47.0)	49 (59.8)	48 (52.7)	56 (52.8)	
**Stages**												
I	6 (75.0)	27 (81.8)	24 (85.7)	28 (80.0)	40 (87.0)	53 (88.3)	57 (89.1)	66 (91.7)	72 (93.5)	73 (89.0)	77 (90.6)	**0.90 (0.022)**
II	0 (0.0)	4 (12.1)	3 (10.7)	1 (2.9)	3 (6.5)	3 (5.0)	4 (6.3)	5 (6.9)	4 (5.2)	5 (6.1)	3 (3.5)	
III-IV	2 (25.0)	2 (6.1)	1 (3.6)	6 (17.1)	3 (6.5)	4 (6.7)	3 (4.6)	1 (1.4)	1 (1.3)	4 (4.9)	5 (5.9)	
**Tumor size**												
<1 cm	3 (50.0)	6 (20.7)	2 (8.0)	11 (32.3)	13 (30.2)	23 (41.8)	22 (37.3)	34 (49.3)	29 (38.7)	27 (34.6)	20 (25.6)	0.96 (0.142)
1.1–1.9 cm	1 (16.7)	6 (20.7)	10 (40.0)	5 (14.7)	6 (13.9)	8 (14.5)	8 (13.6)	17 (24.6)	13 (17.3)	20 (25.6)	20 (25.6)	
2.0–3.9 cm	2 (33.3)	11 (37.9)	4 (16.0)	15 (44.1)	16 (37.2)	15 (27.3)	18 (30.5)	11 (15.9)	21 (28.0)	19 (24.4)	26 (33.3)	
≥4.0 cm	0 (0.0)	6 (20.7)	9 (36.0)	3 (8.8)	8 (18.6)	9 (16.4)	11 (18.6)	7 (10.1)	12 (16.0)	12 (15.4)	12 (15.4)	
**Tumor type**												
PTC	22 (88.0)	45 (93.7)	32 (88.9)	38 (82.6)	70 (87.5)	75 (91.5)	78 (94.0)	78 (94.0)	79 (96.3)	84 (92.3)	101 (95.3)	**1.11 (0.021)**
Other	3 (12.0)	3 (6.3)	4 (11.1)	8 (17.4)	10 (12.5)	7 (8.5)	5 (6.0)	5 (6.0)	3 (3.7)	7 (7.7)	5 (4.7)	
**PTC subtype**												
Classical	13 (61.9)	29 (69.1)	28 (93.3)	27 (77.1)	54 (84.4)	38 (62.3)	40 (71.4)	34 (66.7)	43 (68.3)	57 (80.3)	45 (73.8)	1.01 (0.962)
Follicular	7 (33.3)	11 (26.2)	2 (6.7)	7 (20.0)	8 (12.5)	21 (34.4)	13 (23.2)	15 (29.4)	16 (25.4)	10 (14.1)	14 (22.9)	
**RAI use**												
No	0 (0.0)	2 (4.4)	1 (2.78)	6 (13.3)	8 (10.1)	12 (15.0)	17 (21.5)	22 (28.9)	27 (34.6)	30 (34.5)	40 (39.6)	**0.75 (<0.001)**
Yes	25 (100.0)	43 (95.6)	35 (97.2)	39 (86.7)	71 (89.9)	68 (85.0)	62 (78.5)	54 (71.1)	51 (65.4)	57 (65.5)	61 (60.4)	
**Surgical management**												
Hemithyroidectomy	0 (0.0)	1 (2.1)	0 (0.0)	0 (0.0)	2 (2.5)	4 (4.9)	6 (7.2)	7 (8.4)	3 (3.7)	3 (3.3)	10 (9.5)	0.89 (0.054)
Thyroidectomy	25 (100.0)	47 (97.9)	36 (100.0)	46 (100.0)	78 (97.5)	78 (95.1)	77 (92.8)	76 (91.6)	79 (96.3)	88 (96.7)	95 (90.5)	

OR: Odds Ratio, RAI: Radioactive Iodine.

P-values are estimated for Exact logistic regression and ordinal logistic regression.

### 3.3 Trend in surgery and RAI treatment

Total thyroidectomy showed a modest decline (p = 0.409) from 100% in 2008 to 90.1% in 2018. The RAI treatment showed a significant decline (<0.001) from 100.0% in 2008 to 60.4% in 2018 (Table 2).

### 3.4 Correlates of surgery and RAI treatment

In multiple logistic regression, non-UAE national patients were more likely to require total thyroidectomy (OR: 2.46 [CI: 1.21–4.94]). No other significant correlates of surgical extent were identified (Table 3).

**Table 3:** Simple and multiple logistic regression analysis of surgical management of thyroid cancer with selected variables.

Variables	With Total Thyroidectomy	Unadjusted Analysis		Adjusted Analysis	
	n (%)	OR (95% CI)	P-value	AOR (95% CI)	P-value
Year					
2008–2010	107 (98.2)	Reference		Reference	
2011–2013	195 (93.7)	0.28 (0.06–1.26)	0.098	1.06 (0.10–10.93)	0.962
2014–2016	230 (92.7)	0.24 (0.05–1.05)	0.058	0.33 (0.04–2.83)	0.314
2017–2018	175 (92.6)	0.23 (0.05–1.05)	0.058	0.40 (0.05–3.48)	0.407
Age – Mean ± SD	39.5 ± 12.3	0.99 (0.97–1.02)	0.612	1.01 (0.97–1.05)	0.653
Sex					
Female	542 (93.6)	Reference		Reference	
Male	165 (94.3)	1.13 (0.55–2.31)	0.746	0.60 (0.19–1.89)	0.386
Nationality					
UAE	315 (90.5)	Reference		Reference	
Others	392 (96.5)	2.93 (1.54–5.58)	0.001	5.82 (1.85–18.30)	**0.003**
PTC Subtype					
Other	22 (95.7)	Reference		Reference	
Classical	388 (95.6)	0.98 (0.13–7.68)	0.985	1.14 (0.13–10.45)	0.907
Follicular	117 (94.3)	0.76 (0.09–6.49)	0.802	1.01 (0.10–9.88)	0.992
Tumour size					
≤1.0 cm	166 (88.8)	Reference		Reference	
1.1–1.9 cm	111 (95.7)	2.81 (1.03–7.66)	0.044	2.05 (0.55–7.69)	0.285
2.0–3.9 cm	148 (94.3)	2.08 (0.92–4.68)	0.077	1.43 (0.45–4.51)	0.540
≥4.0 cm	85 (95.5)	2.69 (0.89–8.08)	0.078	1.99 (0.45–8.75)	0.362
Lymph node					
No	384 (91.4)	Reference		Reference	
Yes	156 (97.5)	3.66 (1.28–10.44)	0.015	2.74 (0.74–10.15)	0.131

AOR: Adjusted Odds Ratio, CI: Confidence Interval, OR: Odds Ratio, PTC: Papillary Thyroid Cancer, RAI: Radioactive Iodine, SD: Standard Deviation, UAE: United Arab Emirates.

Multiple logistic regression analysis showed that compared to 2008–2010, the likelihood of receiving RAI was 58% less in 2011–2013, 84% less in 2014–2016, and 92% less in 2017–2018. In addition, non-UAE nationals, those with larger tumor sizes, and LN involvement were more likely to receive RAI treatment (Table 4).

**Table 4:** Simple and multiple logistic regression analysis of RAI with selected co-variates.

Variables	With RAI	Unadjusted Analysis		Adjusted Analysis	
	n (%)	OR (95% CI)	P-value	AOR (95% CI)	P-value
Year					
2008–2010	103 (97.2)	Reference		Reference	
2011–2013	178 (87.3)	0.20 (0.06–0.67)	0.010	0.42 (0.11–1.52)	0.187
2014–2016	167 (71.7)	0.07 (0.10–0.24)	<0.001	0.16 (0.05–0.54)	**0.003**
2017–2018	118 (62.8)	0.05 (0.02–0.16)	<0.001	0.08 (0.02–0.29)	**<0.001**
Age – Mean ± SD	39.5 ± 12.6	0.99 (0.98–1.01)	0.172	0.99 (0.98–1.02)	0.950
Sex					
Female	423 (75.8)	Reference		Reference	
Male	143 (82.7)	1.52 (0.98–2.36)	0.061	1.15 (0.64–2.05)	0.650
Nationality					
UAE National	243 (73.2)	Reference		Reference	
Other national	323 (80.9)	1.56 (1.10–2.21)	0.013	1.63 (1.04–2.56)	**0.033**
Diagnosis					
Others	39 (67.2)	Reference		Reference	
PTC	527 (78.3)	1.76 (0.99–3.13)	0.056	1.82 (0.62–5.38)	0.276
Tumour size					
≤1.0 cm	99 (54.1)	Reference		Reference	
1.1–1.9 cm	81 (73.0)	2.29 (1.38–3.81)	0.001	2.22 (1.26–3.89)	**0.005**
2.0–3.9 cm	130 (85.0)	4.80 (2.82–8.15)	<0.001	4.66 (2.59–8.41)	**<0.001**
≥4.0 cm	78 (92.9)	11.03 (4.58–26.59)	<0.001	9.82 (3.68–26.18)	**<0.001**
Stage					
I	367 (72.7)	Reference		Reference	
II	30 (90.9)	3.76 (1.13–12.52)	0.031	0.77 (0.19–3.19)	0.715
III-IV	22 (71.0)	0.92 (0.41–2.04)	0.836	0.58 (0.10–3.25)	0.633
Lymph node					
No	280 (68.3)	Reference		Reference	
Yes	135 (87.7)	3.30 (1.95–5.57)	<0.001	3.55 (1.86–6.78)	**<0.001**

AOR: Adjusted Odds Ratio, CI: Confidence Interval, OR: Odds Ratio, PTC: Papillary Thyroid Cancer, RAI: Radioactive Iodine, SD: Standard Deviation, UAE: United Arab Emirates.

## 4 Discussion

PTC represented the most common type of TC (92.1%) in our study with the majority having a classical subtype. Follicular TC was found in 4.6% of the patients while anaplastic and medullary cancers were rare. These findings are consistent with studies from the MENA region as well as other parts of the world [[3], [4], [5], [6], [7], [8], [9]]. The mean age at diagnosis of TC in our study was 39.7 years which is lower compared to the age of 44.7 years from a large retrospective study of 12, 508 TC patients diagnosed between 1972 and 2014 from 8 cancer registries in southeast china [19]. It is also lower that the mean age of 48 years from another retrospective study of 77, 276 TC patients diagnosed in the period of 1974–2013 using the surveillance, epidemiology and end results (SEER) cancer registry database in USA [20] or even from our same institution when 135 TC patients were studied between 1991 and 2005 [10]. However, the age at diagnosis was similar to data from a retrospective study of 600 TC patients diagnosed between 2004 and 2005 in nearby Saudi Arabia [7].

The proportion of patients with PTC subtype has increased during the study period, which is consistent with global data, although with a lower magnitude [20,21]. The proportion of advanced stage TC, however, decreased over the study period, which might be partly explained by the increased detection of localized TC cases. The later has been largely ascribed to the widespread use of imaging modalities to diagnose thyroid disorders and or other complaints from surrounding neck structures as well as the increased utilization of fine-needle aspiration to ascertain the nature of thyroid nodules [22,23]. This could also explain the increase in the annual number of TC evaluated in our centre. However, no conclusions can be drawn regarding the temporal trend of TC, as our analysis was based on the absolute number of cases instead of rates.

The 2015 ATA guidelines suggest either lobectomy or total thyroidectomy for the management of TC for tumors 1–4 cm in size [2]. However, there is no universal consensus on the optimal surgical extent in TC management and significant variations are observed worldwide [24]. In our study, the majority of patients underwent total thyroidectomy (93.8%) with a small increase in hemi-thyroidectomy toward the last few years of the study. This finding is similar to another study retrospectively analyzing 44,537 TC patients from SEER database between 2000 and 2014 in US and documenting a lower utilization of lobectomy in low-risk TC [25]. Of interest, cheng ***et al*** showed in 717 TC patients undergoing thyroidectomy between 2008 and 2016 that 44% of those initially eligible for lobectomy would need a completion surgery due to the presence of adverse histopathological features [26]. Similarly, kluijfohout ***et al*** evaluated 1000 patients with TC (size 1–4 cm) who underwent total thyroidectomy between 2000 and 2010 and reported that 122 out of 287 (43%) of patients who are eligible for lobectomy based on the 2015 ATA guidelines would need completion surgery due to the presence of high-risk features such as lymph node involvement (18%) and angioinvasion (12%) [27]. Therefore, determining the extent of surgery continues to be controversial with patient's preference and surgical expertise are paramount in the decision-making process. This issue is more apparent in microPTC, which represented about a quarter of our cohort where all patients underwent surgery. This is in variance with the growing evidence supporting active surveillance as a viable option in the majority of those patients [[28], [29], [30]].

Traditionally, RAI was administered to a large proportion of TC patients following total thyroidectomy. Subsequently, several but not all studies showed a limited benefit of RAI in low-to intermediate-risk groups in reducing disease-related recurrence and or mortality [31,32]. Therefore, the decline in the use of RAI has been advocated for by many **TC** management guidelines. Consistent with this, we noted a substantial decline in the use of RAI from 100% in 2008 to 60% in 2018. Similarly, Park ***et al*** showed a decline in RAI administration in 2015 compared to 1999 mostly in patients with localized disease [4]. Moreover, Sia ***et al*** reported a decline in RAI use from 76.6% in 2002–2006 to 26.8% in 2017–2018 mostly in low-risk patients [5]. The majority of our patients had tumor of <2 cm in size with a limited number presenting with advanced disease (1.9% Stage IV), suggesting overuse of RAI. However, data on RAI indication in our study was not captured, therefore, precluding firm conclusions. Additionally, variation in RAI use is well reported in many countries such as the US and Canada [33,34]. Of interest, the use of RAI for example in T1 disease with unknown or negative lymph node status varied between 15 and 83% among different centers in Canada [34]. This wide variation in RAI use underscores the limited data and uncertainty in the management of a significant proportion of TC patients, necessitating the need for high-quality studies with long term follow up data to confidently guide management in such patients.

The odds of undergoing total thyroidectomy and or receiving RAI ablation were higher in non- UAE nationals. This finding of ethnic variation in management of TC is intriguing and not well reported in the literature. It might stem from the perceived risk of aggressive disease in certain ethnicities. Lo ***et al*** retrospectively evaluated 723 patients with TC from Philippines with 5 years mean follow up and reported higher frequency of aggressive disease at presentation as well as higher recurrence risk [35]. Furthermore, another study from the USA reported a higher rate of adverse histology (microscopic extrathyroidal extension) in Chinese immigrants compared to the non-Asian population [13]. Moreover, Tang ***et al*** retrospectively analyzed 70,346 patients with TC from SEER database between 2004 and 2014 and showed worse overall prognosis for black Americans with **TC** compared to white Americans [14]. Another factor explaining this racial difference in therapy might relate to the uncertainty in establishing long-term follow-up plans in our country as 90% of the populations are non-nationals and many of whom are workers with temporary living plans, which tempts the treating physician to adopt an initial aggressive management strategy [36]. It would be interesting to explore which ethnicity had advanced disease and or received aggressive therapy, but this information was not captured in this study. Additional larger studies with long-term follow-up outcome data would be important to clarify disease behaviour among different ethnicities.

Lymph node involvement in patients with TC is associated with increased recurrence risk and mortality [37]. Therefore, it is not surprising for lymph node involvement to be a predictor for RAI ablation as seen in our study. Tumour size was not associated with the extent of surgery in our study, perhaps due to the low number of patients undergoing lobectomy. However, it was associated with the need for RAI ablation. In 2015 ATA guidelines, tumor size is not an indication for RAI ablation in the absence of other adverse features [2]. Nonetheless, it is reassuring that the use of RAI has declined steadily during the study.

Our study has strengths and limitations. To our knowledge, this is the first study in the MENA region to describe trends in TC characteristics and management patterns following publications of professional TC management guidelines using a large number of patients. The main limitations of the study relate to its retrospective nature with incomplete documentation of data that could alter the decision of surgery and RAI such as the family history of TC, radiation exposure, and the number and size of involved lymph nodes. In addition, the reasons for determining the extent of surgery and the need/dose of RAI were not consistently recorded. Also, this study reflects the experience from a large referral center and may not be generalizable to other centers in the country.

## 5 Conclusion

In summary, there is a substantial increase in the annual number of TC patients evaluated in our centre with the majority having PTC and stage 1 disease at diagnosis. Nationality and LN involvement were independent predictors of total thyroidectomy, while the year of diagnosis, nationality, LN involvement, and tumor size were predictors of RAI use. **More** studies are **needed** to understand factors affecting variation in **TC** management in UAE.

## Funding source

Bayer Middle East FZE supported this manuscript publication fees. The funder had no role in study design, data collection and analysis or preparation or approval of the manuscript.

## Author contributions

M.A., A.S., A.M., E.A., A.K.M. conceived and wrote the manuscript. A.F. performed the statistical analysis. **A.F.,** S.R., A.O.H. revised the manuscript for important intellectual content. All authors reviewed and approved the final manuscript.

## Ethical approval

The study was approved by Al Ain Medical District Human Research Ethical Committee and consent was waived.

## Consent

The study was approved by Al Ain Medical District Human Research Ethical Committee and consent was waived.

## Trial registry number

1. Name of the registry: Chinese Clinical Trial Registry.

2. Unique Identifying number or registration ID: ChiCTR2100043249.

3. Hyperlink to your specific registration (must be publicly accessible and will be checked): http://www.chictr.org.cn/showprojen.aspx?proj=121453.

## Guarantor

Dr. Malik Azhar.

Dr. Khaled Aldahmani.

## Provenance and peer review

Not commissioned, externally peer-reviewed.

## Declaration of competing interest

The authors have no conflicts of interest to declare.
